# Supplementary material for: Timing along the cardiac cycle modulates neural signals of reward-based learning
Source: Nat Commun. 2024 Apr 6;15:2976. doi: 10.1038/s41467-024-46921-5 (PMC10998831; doi:10.1038/s41467-024-46921-5)
Supplement: Supplementary file 1 — Supplementary information [file 41467_2024_46921_MOESM1_ESM.pdf]

SUPPLEMENTARY FIGURES

Supplementary Figure 1: Model comparison, model fit and parameter recovery

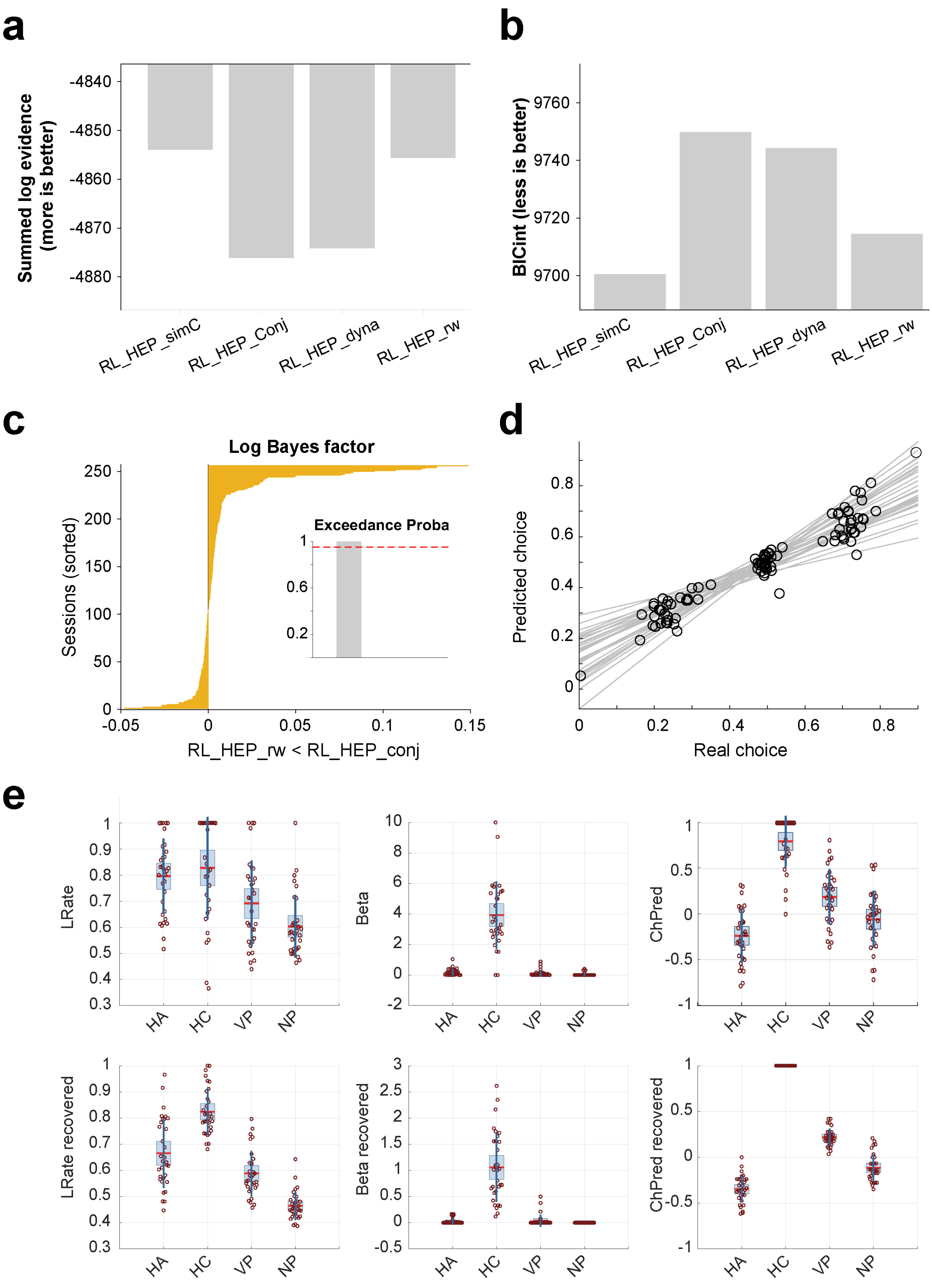

Supplementary Figure 1. (**a**) Summed log-model evidence using a variational inversion scheme for all models. Higher scores indicate better fit. (**b**) Nested model comparison using the summed integrated Bayesian Information Criterion (BICint) scores for all models. Lower scores indicate better fit.

RL_HEP_SimC is the winning model. (**C**) Comparisons of Bayes factors between best and second-best models. Bars are individual blocks sessions and direction of the bars (left vs right) indicate better model fit in favour of the RL_HEP_SimC (going to the right) compared to RL_HEP_rw (going to the left). (**Small panel**) Exceedance probability also favours the SimC model among our set of candidate models. The dashed red line indicates an exceedance probability of 0.95. (**d**) Model-predicted choice probabilities (y-axis) derived from the SimC algorithm (binned into four bins – bin size of 0.25 - and averaged across all subjects and across symbols) closely matched participants observed behavioural choices (x-axis), calculated for each bin as the fraction of trials in which they chose one colour. (**e**) Parameters fitted (top row) and recovered (bottom row) following parameter recovery procedure. The box plots present the mean, the SEM and 95% confidence intervals for the mean. Jittered raw data are plotted for each group.

Supplementary Figure 2: Individual Machine Learning Performance

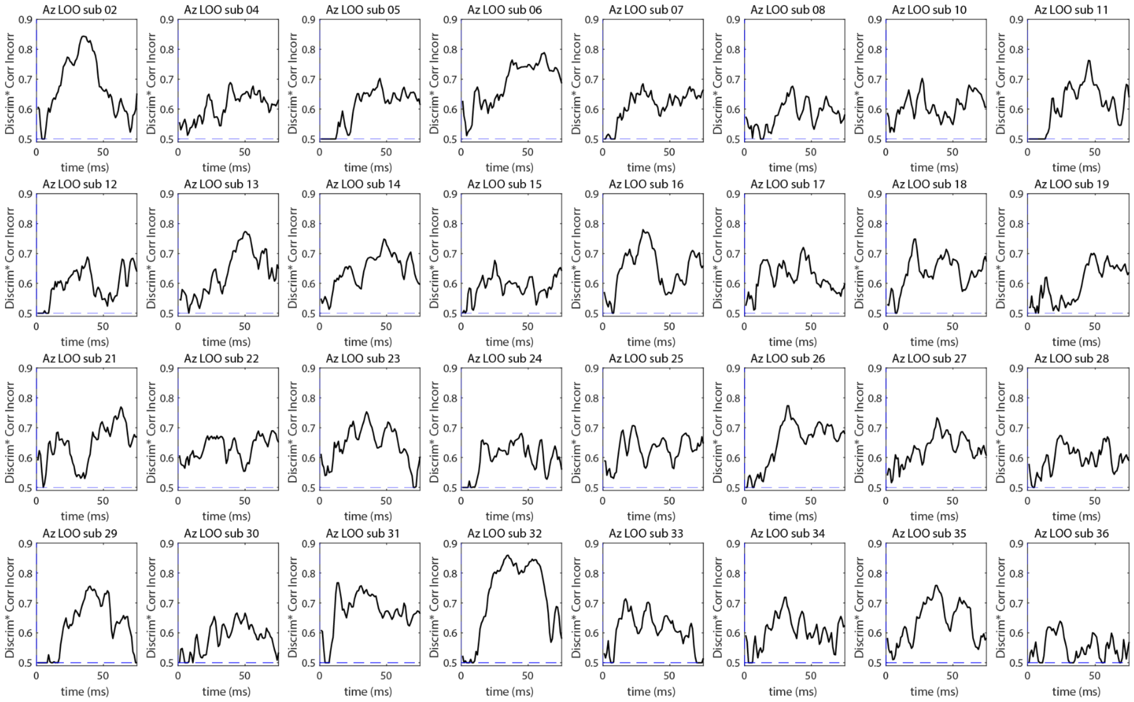

Supplementary Figure 2. Discriminator performance following Leave-One-Out procedure (Az) during high-vs-low salient outcome discrimination of HEP-locked EEG data, for all subjects.

Supplementary Figure 3: Control analyses for potential unaccounted effects

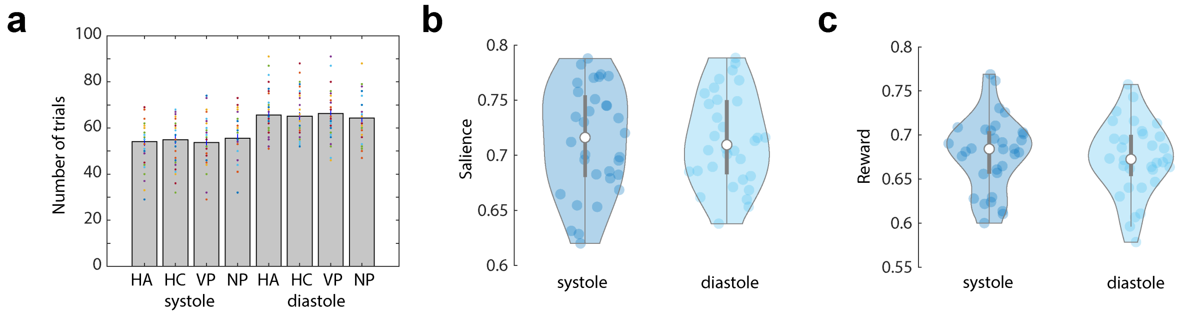

Supplementary Figure 3. Control analyses for potential unaccounted effects. **(a)** The average number of trials for systole and diastole was the same across predictive and non-predictive blocks. **(b)** Mean absolute PE difference between the HEP for all outcomes presented at systole versus diastole (N=32). A violin plot is used to present all participants' average. **(c)** Mean reward difference between the HEP for all outcomes presented at systole versus diastole (N=32). Neither the absolute PE (from the RL model) nor the reward was different between the systole and diastole outcomes. A violin plot is used to present all individual participants' averages, as well as the mean and SEM in **b-c**.

Supplementary Figure 4: HEP morphology and replication of main figure 2 with the first HEP only

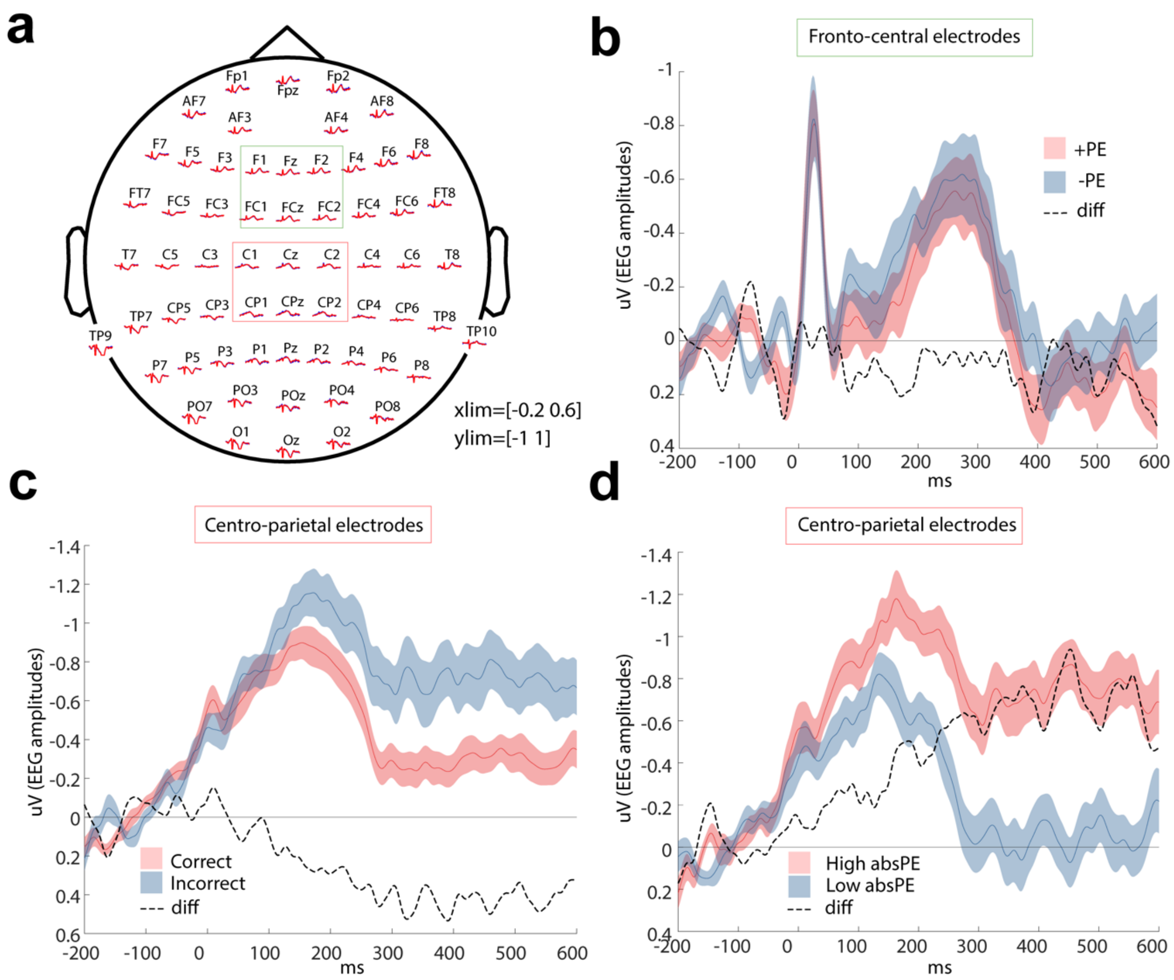

Supplementary Figure 4. HEP morphology and results. **(a)** Grand average waveforms across the scalp time-locked to the onset of the R-wave which is the biggest electrical wave generated during normal conduction (time 0ms, see Methods). The set of electrodes clustered by ROIs (colour-coded) for the fronto-central and central-parietal electrodes are represented for further analyses **(b-d)** HEP waveforms across all trials following the onset of the R-wave (at time 0 ms) are shown separately (only the first HEP after feedback). This is presented for positive and negative signed prediction errors (PEs) for the frontal cluster in **(b)**; correct vs incorrect outcome in **(c);** and for high and low surprising outcomes (absolute PEs) in **(d)**. The dotted line represents the difference between the conditions (represented in red and blue).

Supplementary Figure 5: Control analyses for potential unaccounted effects of outcome (valence and absolute PE) in the relationship between HEP and heart cycle

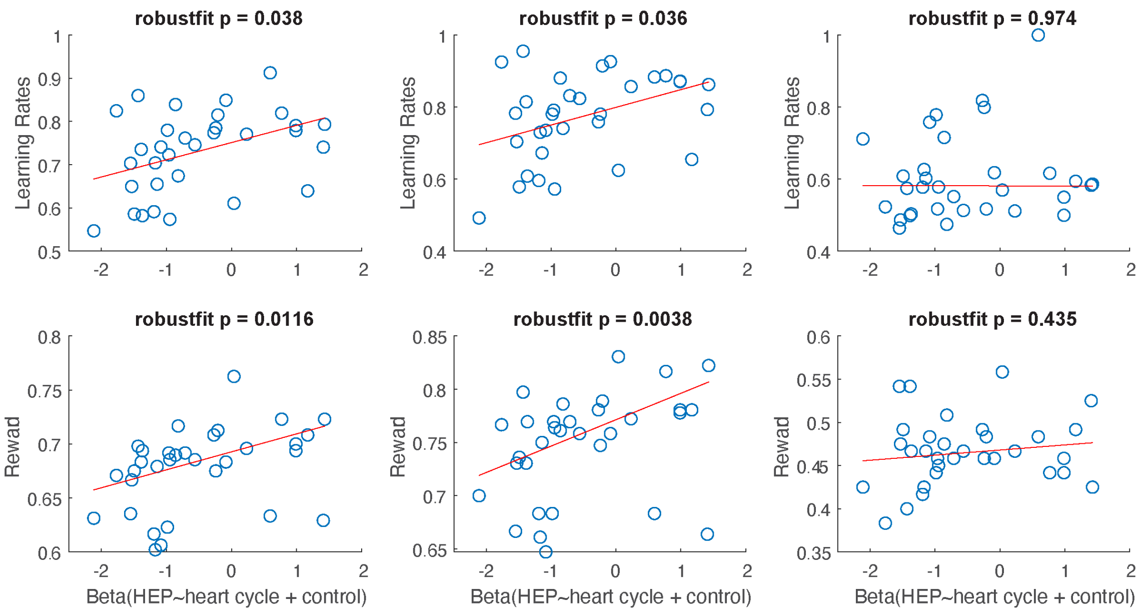

Supplementary Figure 5. Control analyses for potential unaccounted effects of outcome (valence and absolute PE) in the relationship between HEP and heart cycle. Results of the correlation between the regression coefficient for each participant between absPE-HEP and systole/diastole and the mean reward and learning rates in the task. In red, the fit of the robust regression. Any of these results remain true even when including a covariate indexing features of the external outcome type – reward and absolute PE from the model. Particularly, in learning rates – all task blocks learning rates (top left) – predictive blocks learning rates (top middle) – non predictive blocks reward (top right) – all task blocks reward (bottom left) – predictive blocks reward (bottom middle) – non predictive blocks reward (bottom right). We did not adjust the p-value for multiple comparison.

SUPPLEMENTARY TABLES

Supplementary Table 1: Parameter estimates and parameter recovery

**Parameter estimate for best behavioural model, depicted as mean ± SD and Recovery parameters estimates (across subjects) ± SD. Parameters are presented below the ones found during the fitting procedure.**				
	HC	HA	VP	NP
Learning rate	0.795 ± 0.144	0.827 ± 0.196	0.691 ± 0.164	0.603 ± 0.120
	0.665 ± 0.132	0.824 ± 0.089	0.587 ± 0.086	0.464 ± 0.053
Correlation	0.4324 ± 0.2758			
SoftMax inv. Temp	0.199 ± 0.220	3.927 ± 2.203	0.119 ± 0.216	0.038 ± 0.105
	0.032 ± 0.057	1.057 ± 0.658	0.038 ± 0.113	0.000 ± 0.000
Correlation	0.8259 ± 0.2707			
Choice stickiness	-0.235 ± 0.294	0.798 ± 0.280	0.190 ± 0.301	-0.054 ± 0.312
	-0.348 ± 0.147	-1.00 ± 0.0003	-0.219 ± 0.095	-0.115 ± 0.149
Correlation	0.7242 ± 0.2153			

Supplementary table 1. Recovery parameters estimates (across subjects) ± SD. Parameters are presented below the ones found during the fitting procedure. Correlations are presented between fitted and recovered parameters across blocks types and participants.

Supplementary Table 2: Mixed effect model results

Name	Estimate	SE	tStat	DF	pValue	Lower	Upper
Intercept	0.329	0.008	40.807	13877	0.000	0.313	0.345
SysDias	-0.022	0.012	-1.889	13877	0.059 .	-0.046	0.001
STV	0.024	0.010	2.415	13877	0.016 *	0.004	0.043
AbsPE	0.112	0.008	13.788	13877	< 0.001 ***	0.096	0.128
SysDias: STV	-0.007	0.014	-0.461	13877	0.645	-0.035	0.022
SysDias: AbsPE	0.025	0.012	2.106	13877	0.035 *	0.002	0.049
STV: AbsPE	-0.017	0.010	-1.723	13877	0.085 .	-0.037	0.002
SysDias: STV: AbsPE	-0.003	0.015	-0.202	13877	0.840	-0.032	0.026

Supplementary Table 2. Results of the mixed-effect model. SE indicates Standard Error. Tstats indicates t-statistics. DF indicates degree of freedom. '.' Indicates $p<0.1$, '*' Indicates $p<0.05$, '***' Indicates $p<0.001$. Lower and upper refer to confidence intervals.
