# Supplementary material for: Timing along the cardiac cycle modulates neural signals of reward-based learning
Source: Nat Commun. 2024 Apr 6;15:2976. doi: 10.1038/s41467-024-46921-5 (PMC10998831; doi:10.1038/s41467-024-46921-5)
Supplement: Supplementary file 3 — Reporting Summary [file 41467_2024_46921_MOESM3_ESM.pdf]

nature portfolio

Corresponding author(s): Elsa Fouragnan

Last updated by author(s): 22/12/2023

Reporting Summary

Nature Portfolio wishes to improve the reproducibility of the work that we publish. This form provides structure for consistency and transparency in reporting. For further information on Nature Portfolio policies, see our Editorial Policies and the Editorial Policy Checklist.

Statistics

For all statistical analyses, confirm that the following items are present in the figure legend, table legend, main text, or Methods section.

n/a	Confirmed	
☐	☒	The exact sample size (*n*) for each experimental group/condition, given as a discrete number and unit of measurement
☐	☒	A statement on whether measurements were taken from distinct samples or whether the same sample was measured repeatedly
☐	☒	The statistical test(s) used AND whether they are one- or two-sided<br>*Only common tests should be described solely by name; describe more complex techniques in the Methods section.*
☐	☒	A description of all covariates tested
☐	☒	A description of any assumptions or corrections, such as tests of normality and adjustment for multiple comparisons
☐	☒	A full description of the statistical parameters including central tendency (e.g. means) or other basic estimates (e.g. regression coefficient) AND variation (e.g. standard deviation) or associated estimates of uncertainty (e.g. confidence intervals)
☐	☒	For null hypothesis testing, the test statistic (e.g. *F*, *t*, *r*) with confidence intervals, effect sizes, degrees of freedom and *P* value noted<br>*Give P values as exact values whenever suitable.*
☐	☒	For Bayesian analysis, information on the choice of priors and Markov chain Monte Carlo settings
☐	☒	For hierarchical and complex designs, identification of the appropriate level for tests and full reporting of outcomes
☐	☒	Estimates of effect sizes (e.g. Cohen's *d*, Pearson's *r*), indicating how they were calculated

Our web collection on statistics for biologists contains articles on many of the points above.

Software and code

Policy information about availability of computer code

Data collection	Stimuli and responses were presented and collected using Presentation software (Neurobehavioral Systems Inc., Albany, CA). EEG was recorded with sintered Ag/AgCl electrodes from 64 scalp electrodes mounted equidistantly on an elastic electrode cap (64Ch-Standard-BrainCap for TMS with Multitrodes; EasyCap; two cap sizes, 56 cm and 58 cm head circumference).
Data analysis	Off-line EEG analysis was performed using Fieldtrip (https://www.fieldtriptoolbox.org/). Detection of R-peaks in the ECG recording was done using the Pan-Tompkins algorithm as implemented in MATLAB (https://www.researchgate.net/publication/313673153_Matlab_Implementation_of_Pan_Tompkins_ECG_QRS_detect). Physiological artefacts such as eye blinks, saccades and the volume-conducted cardiac-field artifact (CFA) were corrected by means of independent component analysis (RUNICA, logistic Infomax algorithm) as implemented in the FieldTrip toolbox. We adopted a non-parametric, cluster-based permutation approach to first determine the HEP morphology, and then estimate any HEP amplitude modulation as a function of learning. Subject-wise activation time-courses were extracted and passed to the statistical analysis procedure in FieldTrip. The multivariate analysis and reinforcement learning fitting required custom scripts which can be find here: https://github.com/efouragnan/EEG-CRS_learning.

For manuscripts utilizing custom algorithms or software that are central to the research but not yet described in published literature, software must be made available to editors and reviewers. We strongly encourage code deposition in a community repository (e.g. GitHub). See the Nature Portfolio guidelines for submitting code & software for further information.

Data

Policy information about availability of data

All manuscripts must include a data availability statement. This statement should provide the following information, where applicable:

- Accession codes, unique identifiers, or web links for publicly available datasets
- A description of any restrictions on data availability
- For clinical datasets or third party data, please ensure that the statement adheres to our policy

We have deposited all choice raw data in an OSF repository. All reinforcement learning results in this paper are derived from these data alone. We have also deposited all neural data presented in the manuscript in the same OSF repository. The accession code is: https://osf.io/qgw7h/

Human research participants

Policy information about studies involving human research participants and Sex and Gender in Research.

Reporting on sex and gender	Gender and biological sex were determined based on self-reporting. 32 participants were included in the analyses from which 10 were identified as female and 22 identified as male. Consent has been obtained for sharing this information. No sex and gender based analyses were performed .
Population characteristics	Thirty-five healthy, right-handed adults participated in the experiment. 3 participants were excluded due to excessive noise in the EEG signal so that data from 32 participants were included in the analyses (24 ± 7.13; 10; 0.83 ± 0.13; where numbers correspond to mean age ± SD; number of female participants, handiness mean ± SD; as measured by the Edinburgh handedness inventory). Gender and biological sex were determined based on self-reporting. All participants were naïve to the task, had no personal or familial history of neurological or psychiatric disease, were right-handed, gave written informed consent (Medical Science Interdivisional Research Ethics Committee, Oxford RECC, No. R55856/RE002), and received monetary compensation for their participation.
Recruitment	Participants were recruited through general advertisement, study flyers, existing database of priors participants, emails disseminated through the community and word to mouth. Participants received monetary compensation for their participation. We do expect self selection bias.
Ethics oversight	The study received ethical approval which was obtained through the Medical Science Interdivisional Research Ethics Committee of Oxford with reference number: Oxford RECC, No. R55856/RE002.

Note that full information on the approval of the study protocol must also be provided in the manuscript.

Field-specific reporting

Please select the one below that is the best fit for your research. If you are not sure, read the appropriate sections before making your selection.

☒ Life sciences ☐ Behavioural & social sciences ☐ Ecological, evolutionary & environmental sciences

For a reference copy of the document with all sections, see nature.com/documents/nr-reporting-summary-flat.pdf

Life sciences study design

All studies must disclose on these points even when the disclosure is negative.

Sample size	Sample sizes were determined based on power analyses and previous studies that have used similar reward learning paradigms to investigate brain responses during learning (Fouragnan et al., 2015; 2017), and studies that have measured the Cardiac Related Signal to investigate neural responses to heartbeats in humans (Sel et al., 2017).
Data exclusions	3 participants were excluded due to excessive noise in the EEG signal.
Replication	The experiment was perform only once.
Randomization	There was only one condition. We did randomize the order of blocks within participants to account for a potential order effect.
Blinding	Investigators were not blinded as there was only a single condition performed.

Reporting for specific materials, systems and methods

We require information from authors about some types of materials, experimental systems and methods used in many studies. Here, indicate whether each material, system or method listed is relevant to your study. If you are not sure if a list item applies to your research, read the appropriate section before selecting a response.

Materials & experimental systems

n/a	Involved in the study
☒	☐ Antibodies
☒	☐ Eukaryotic cell lines
☒	☐ Palaeontology and archaeology
☒	☐ Animals and other organisms
☒	☐ Clinical data
☒	☐ Dual use research of concern

Methods

n/a	Involved in the study
☒	☐ ChIP-seq
☒	☐ Flow cytometry
☒	☐ MRI-based neuroimaging
